# Burnout among Residents at the Mohammed VI University Hospital of Marrakech

**DOI:** 10.1192/j.eurpsy.2026.11488

**Published:** 2026-07-31

**Authors:** H. Zizi, R. Rahoua, Z. Ennaciri, I. Adali, F. Manoudi

**Affiliations:** Ibn Nafis hospital, Psychiatry, Mohammed VI university hospital, Marrakech, Morocco

## Abstract

**Introduction:**

Burnout syndrome is a contemporary condition representing the ultimate stage of work-related stress. It particularly affects professions requiring intense personal involvement, such as healthcare workers, with an even greater severity among medical residents.

**Objectives:**

The aim of this study was to determine the prevalence of burnout among medical residents at Mohammed VI University Hospital of Marrakech and to identify its risk factors.

**Methods:**

We conducted a cross-sectional descriptive and analytical study on a sample of 200 medical residents at Mohammed VI University Hospital of Marrakech during February 2025. Burnout was assessed using the Maslach Burnout Inventory (MBI). Data were collected through a self-administered questionnaire. Data entry and analysis were performed using SPSS software.

**Results:**

Our study sample consisted of 63% women. The most common age group was 25–30 years (48.4%), and 69.2% of residents were single. The most frequent personal psychiatric histories were anxiety disorders (27.3%) and depression (26.4%). Suicide attempts accounted for 2.7%. Regarding family psychiatric history, depression was the most common (16.7%). Medical specialties represented 69.7% of the sample. First- and second-year residents accounted for 63.7%. About 56.8% of residents worked between 40 and 60 hours per week, with a mean of 5 night shifts, ranging from 0 to 15. According to the MBI, 35% had high emotional exhaustion, 38% high depersonalization, and 55% low personal accomplishment. Severe burnout was observed in 21% of residents. This study identified significant associations between severe burnout and having more than 5 monthly night shifts (p=0.03). Severe burnout was also associated with the desire for career change (p=0.03). Leisure activities significantly reduced emotional exhaustion and increased personal accomplishment, while choosing medical studies out of personal conviction was a protective factor against burnout.

**Conclusions:**

This study revealed an alarming prevalence of burnout among medical residents at Mohammed VI University Hospital of Marrakech. Preventive measures should be implemented to preserve the mental health of this high-risk population.

**Disclosure of Interest:**

None Declared

